# Effect of the High-FODMAP Diet on Bowel Preparation for Colonoscopy: A Multicenter, Prospective Cohort Study

**DOI:** 10.1155/2020/1612040

**Published:** 2020-06-22

**Authors:** Peng Cheng, Ruijun Ma, Shuling Wang, Jun Fang, Zhengrong Zhong, Yu Bai, Xiangjun Meng, Zhaoshen Li

**Affiliations:** ^1^Department of Gastroenterology, Shanghai Ninth People's Hospital, Shanghai Jiao Tong University School of Medicine, Shanghai, China; ^2^Center for Gastrointestinal Endoscopy, Shanxi Provincial People's Hospital, Taiyuan, China; ^3^Department of Gastroenterology, Changhai Hospital, Second Military Medical University, Shanghai, China; ^4^Department of Gastroenterology, Zhongnan Hospital of Wuhan University, Wuhan, China; ^5^Department of Clinical Laboratory, Shanghai Ninth People's Hospital, Shanghai Jiao Tong University School of Medicine, Shanghai, China

## Abstract

**Background:**

Optimal bowel preparation for colonoscopy is fundamental to a successful examination. The FODMAP diet can increase the content of intestinal water and gas, but its impact on bowel cleanliness and bubbles has not been reported. This study was therefore aimed at evaluating the effect of the FODMAP diet on the quality of bowel preparation and the adenoma detection rate (ADR).

**Methods:**

This was a multicenter, prospective cohort study involving consecutive patients who underwent colonoscopy in two centers in China. Patients were assigned to one of two groups: high-FODMAP or nonhigh-FODMAP diet. ODMP Software was used for the identification of FODMAP diet types. The primary outcome was ADR; secondary outcomes were the quality of bowel preparation, measured by the Boston bowel preparation scale and bubble scores.

**Results:**

There were 365 patients included. Patients in the high-FODMAP-diet group showed poor bowel cleansing efficacy: BBPS ≥ 6 in 76.8% vs. 90.3% (*P* < 0.01) and bubble scores of 2.42 ± 1.69 vs. 1.32 ± 1.63 (*P* < 0.001). The intubation time was significantly longer in the high-FODMAP-diet group (7.07 ± 5.18 vs. 5.46 ± 3.05 min; *P* = 0.002). The High-FODMAP diet was an independent risk predictor for inadequate bowel preparation. There were no statistically significant differences in ADR between the two dietary groups.

**Conclusion:**

The high-FODMAP diet significantly reduced the quality of bowel preparation. We recommend the consumption of nonhigh-FODMAP diet in bowel preparation as a reference standard for dietary regimen. This method was effective, flexible, referable, and well tolerated, which could help to provide patients a valuable dietary guidance in bowel preparation.

## 1. Introduction

Colorectal cancer (CRC) contributes significantly to mortality related to cancer worldwide. Recent studies have shown an increase in the incidence of CRC among younger people. In an effort to reduce the risk of death associated with CRC, early identification and removal of adenomatous polyps are crucial [1]. Colonoscopy, at this point, is the best modality for this goal [2]. Optimal bowel preparation for colonoscopy is fundamental to a successful examination. Bowel preparation correlates with adenoma detection rate (ADR), procedure duration, and the need for repeat procedures [2–5]. The presence of fecal residue and bubbles may lower ADR, prolong the procedure duration, and affect patient compliance [6].

Dietary management plays an important role in bowel preparation. Traditionally, patients have been instructed to consume a clear liquid diet (CLD) the day before a colonoscopy, further exacerbating patient noncompliance. In recent years, low-residue/low-fiber diet strategies have been presented and recommended to replace the CLD, appearing to be as effective in terms of the quality of bowel preparation while demonstrating higher patient tolerability and compliance [7–11]. However, what is the “low-residue or low-fiber diet”? To date, no clear definition has been proposed, and no reference has been made as to its type. No study has examined a fully flexible low-residue or low-fiber diet that may be consumed on the day prior to the colonoscopy.

FODMAP stands for fermentable, oligosaccharides, disaccharides, monosaccharides and polyols. It can increase the content of intestinal water and gas [12]. FODMAP has been shown to cause symptoms of abdominal bloating in IBS patients [13–16]. However, its impact on bowel cleanliness and bubbles has not been reported. Therefore, this study was aimed at evaluating the effect of different types of FODMAP diet on the quality of bowel preparation and the adenoma detection rate (ADR). Furthermore, the definition of FODMAP is clear, and there are types of software available to query the FODMAP types in various foods. We imagine that a certain type of FODMAP diet would be both effective in improving the quality of bowel preparation and well tolerated. The results of the study would help to provide patients with flexible, readily available, and accurate precolonoscopy dietary guidance.

## 2. Methods

### 2.1. Study Design

This study was a prospective, multicenter, endoscopist-blinded trial. Data were collected from two tertiary endoscopy units (Shanghai Ninth People's Hospital, Shanghai Jiao Tong University School of Medicine, Shanxi Provincial People's Hospital) from May 2017 to October 2017. In both units, colonoscopy was performed by endoscopists with experience of >1000 colonoscopy procedures. The endoscopes used in this study included CF-H260AI and CF-H290I (Olympus, Japan). No analgesia or sedation was used in any of the patients. Biopsies of suspected polyps and tumors were performed; final diagnosis was based on histopathological examination. In each center, patients were assigned to one of two groups according to the food diary card during the day prior to colonoscopy: high-FODMAP diet or nonhigh-FODMAP diet (participants were asked to complete a food diary card depending on the individual's diet during the day prior to colonoscopy). ODMP Software was used for the identification of different FODMAP diet types. Data on the participants' individual and clinical variables were retrieved from the computerized database. The study protocol was in compliance with the Helsinki Declaration. This study was approved by the Ethics Committee of Changhai Hospital.

### 2.2. Patients

Consecutive adult patients aged between 18 and 75 years and scheduled for outpatient colonoscopy were included. Exclusion criteria were patients with known or suspected toxic megacolon, severe colitis, heart failure, renal failure, severe liver and brain diseases, intestinal obstruction, restricted mobility, history of drug use related to intestinal dynamics (such as opioids, antidepressants, or calcium antagonists), hypersensitivity to any of the ingredients; pregnancy or lactation and those planning to become pregnant; and anyone who had participated in another clinical trial in the previous 60 days. Patients were informed about the aims, procedures, benefits, and likely risks associated with their participation in the study and gave written informed consent prior to their enrollment.

### 2.3. Procedure

In each center, the bowel preparation received by all participants was 3 L polyethylene glycol (PEG) (Wanhe Pharmaceutical Co. Ltd., China) 6–8 hours prior to colonoscopy, with the mixture being orally administered at a dose of 250 mL every 10–15 minutes. On the day prior to colonoscopy, participants were asked to record their diet on a food diary card that was provided beforehand. A technician or nurse who was not involved in the colonoscopy examinations was responsible for assigning patients to the high-FODMAP-diet group or the nonhigh-FODMAP-diet group based on the diet information recorded on the food diary card, and the diet types were identified by ODMP Software.

The participants' height and weight were measured for calculation of body mass index (BMI), and they were requested to complete a questionnaire that was specifically designed for the study and included data on variables such as age, gender, constipation, previous history of abdominal surgery, diabetes, and hypertension. Other quality indicators, including cecal intubation rate, insertion time, and withdrawal time, were also recorded. Insertion time was recorded as the time for insertion of the endoscope from the anus to the cecum. Colonoscopy withdrawal time was recorded as the time from the cecum to the anus but excluded the time for biopsy performance or removal of polyps. These times were recorded by nurses or the endoscopists who performed the colonoscopy. The occurrence of gastrointestinal symptoms (nausea, vomiting, abdominal pain, and bloating) during the process of bowel preparation was also recorded.

### 2.4. Endpoints

The primary endpoints of this study included the Boston bowel preparation scale (BBPS) score, which was developed to evaluate the quality of colon preparation and was used in our previous study [17, 18]. Points (segment score) were assigned as follows: 0, unprepared colon segment; 1, major residual stool or opaque liquid; 2, minor residual staining; and 3, entire mucosa easily visible. Each section of the colon (i.e., the right, the transverse, and the rectosigmoid colon) was rated [19]. High quality of bowel preparation was defined as a total BBPS score ≥ 6. Inadequate bowel preparation was defined as a total BBPS score < 6 [20]. In addition, the bubble score was defined on a 4-point scale according to its impact on mucosal visibility (0, no bubbles; 1, minimal or occasional bubbles (must be actively sought); 2, moderate or obviously present; and 3, severe or so many bubbles that vision is obscured), as has been used in previous studies [21–24]. Scores were assigned by a colonoscopist who was blinded to the group allocation scheme.

Secondary outcome was ADR, defined as the proportion of patients undergoing colonoscopy in whom at least one histologically confirmed colorectal adenoma was detected [17]. Other outcomes were cecal intubation time and withdrawal time.

### 2.5. Statistical Analysis

The chi-squared test was used for categorical data. The mean (± standard deviation (SD)) and range were calculated for continuous variables, and between-group differences were assessed by using Student's *t*-test. Multivariate logistic regression analysis of independent risk factors was used to determine the influence of the high-FODMAP diet, sex, diabetes mellitus, constipation, and complete intake of the medication on inadequate bowel preparation, which were significant on single-factor analysis. *P* values < 0.05 were considered statistically significant. Statistical software from SPSS (Version 20.0, SPSS Inc., Chicago, Illinois, USA) was used for data analysis.

## 3. Results

**Figure 1:**
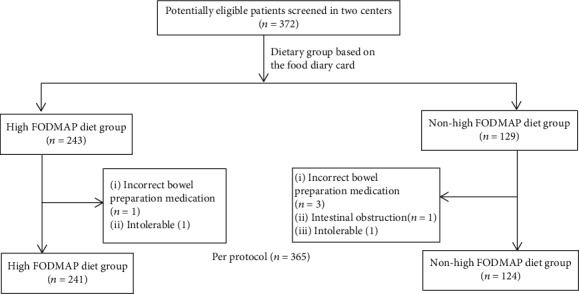
Study flow diagram.

A total of 372 patients were enrolled, and 365 with successful cecal intubation were included in the analysis. The main reason for exclusion included consumption of the incorrect bowel preparation medication (*n* = 4), having intestinal obstruction due to CRC (*n* = 1), and lack of tolerance of the procedure (*n* = 2). A total of 241 patients were assigned to the high-FODMAP-diet group and 124 to the nonhigh-FODMAP-diet group based on the diet information recorded on the food diary card and the diet types identified by ODMP Software. The process of patient screening and exclusion is illustrated in Figure 1. The mean age of the patients was 53.0 ± 12.8 years (range 19-85 years), and 49.0% were men. Their mean BMI was 23.8 ± 3.3 kg/m^2^. Comparisons of the baseline clinical and demographic characteristics of the two subject groups are shown in Table 1.

Comparison of the two dietary groups on colonoscopy procedure-associated parameters is shown in Table 2. The intubation time was significantly longer in the high-FODMAP-diet group (7.07 ± 5.18 minutes vs. 5.46 ± 3.05 minutes, *P* = 0.002). There were no statistically significant differences in terms of withdrawal time or ADR between the two dietary groups.

**Figure 2:**
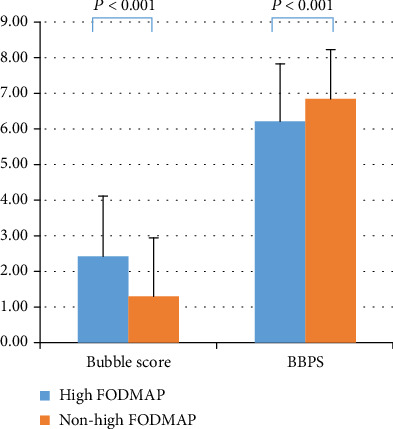
Comparison of bowel cleansing efficacy.

For the primary endpoint, there was a significantly higher score for bubbles and its overall impact on mucosal visibility in the high-FODMAP-diet group than in the nonhigh-FODMAP-diet group (2.42 ± 1.69 vs. 1.32 ± 1.63; *P* < 0.001). The high-FODMAP diet increased bubbles and foam in all colon segments. The BBPS score in the high-FODMAP-diet group was significantly lower than that in the nonhigh-FODMAP-diet group (6.21 ± 1.62 vs. 6.86 ± 1.37; *P* < 0.001) in total and in separate colon segments (Table 3, Figure 2). The high-FODMAP-diet group was significantly lower in terms of quality of bowel preparation (BBPS ≥ 6 in 76.8% vs. 90.3%; *P* < 0.01) (Table 3).

Endoscopy center, sex, age, BMI, dietary group, past history of CRC or polyps, history of abdominal surgery or gallbladder resection, diabetes mellitus (DM), hypertension, constipation, and complete intake of medication were included in the univariate analysis of risk factors for inadequate bowel preparation (Table 4). Sex (*P* = 0.020), dietary group (*P* = 0.002), DM (*P* = 0.009), constipation (*P* = 0.006), and complete intake of the medication (*P* = 0.010) were significantly associated with inadequate bowel preparation. There was no statistical significance with the others.

The results of multivariate logistic regression analysis of independent risk factors that were significant in single factor on inadequate bowel preparation are shown in Table 5. The independent risk predicators for inadequate bowel preparation were shown to be high-FODMAP diet (*P* = 0.002), DM (*P* = 0.010), and constipation (*P* = 0.003), and the independent protective factors were being male (*P* = 0.002) and complete intake of the medication (*P* = 0.009).

## 4. Discussion

High-quality bowel preparation is a prerequisite for successful colonoscopy. Inadequate bowel preparation may result in missed diagnosis, misdiagnosis, and reduced adenoma detection rate (ADR) [25]. Ideally, bowel preparation regimens should be both effective and well tolerated and mainly include dietary regimen and bowel cleaning.

With regard to the diet strategies for bowel preparation, a dietary restriction to clear fluids on the day prior to colonoscopy remains the standard among many institutions. Recently, a low-residue/low-fiber diet has been recommended. Compared with clear liquid diets, studies have confirmed [8–10] that it appears to be as effective with respect to the quality of bowel preparation but demonstrates higher patient tolerability and compliance. However, to date, there has been no scientifically acceptable definition of residue. The impossibility of estimating the amount of residue produced by the digestion of various foods complicates a consensus definition for residue. In the literature, residue mostly refers to any indigestible food substance that remains in the intestinal tract and contributes to stool bulk [26, 27]. This means that the residue is primarily indigestible material, microorganisms, secretions, and cells shed from the digestive tract [28]. Likewise, dietary recommendations regarding the actual composition of low-residue and/or low-fiber diets are different. To date, no clear definition has been proposed for any of these. Cunningham [26] pointed out that all foods produce some gastrointestinal residue. Lijoi et al. [29] described a low-fiber diet as a total dietary fiber intake of <10 g a day. Although these studies defined a low-fiber diet in a quantitative way, no reference has been made to the type of dietary fiber. Other studies have only defined examples of foods that were allowed and not allowed in low-fiber diets [30].

The influence of the FODMAP diet on water and gas in the intestinal tract has been confirmed [12]. The purpose of this study was to determine whether the high-FODMAP diet significantly affected the quality of bowel preparation and can be used as a reference dietary regimen for bowel preparation.

With these issues in mind, the current study was designed. Both groups of patients showed good tolerability and compliance with the dietary regimen because of the absence of dietary restrictions in this study. On comparison of the two dietary groups, there was a significantly higher score for bubbles in the high-FODMAP-diet group than in the nonhigh-FODMAP-diet group, in total and separately in colon segments. In addition, the BBPS score in the high-FODMAP-diet group was significantly lower than that of the other group. The proportion of high-FODMAP diet patients with high-quality bowel preparation was 76.8%, which was significantly lower than that of the nonhigh-FODMAP-diet patients (90.3%). This showed that the high-FODMAP diet not only increased bowel bubbles and foam but also significantly impacted bowel preparation. Comparing the two dietary groups, the intubation time was significantly longer in the high-FODMAP-diet group, as a result of more patients needing washing and suction during colonoscopy. It is well known that repeated washes of the mucosa due to the presence of foam and residue prolong the intubation time.

We showed that sex, dietary group, DM, constipation, and complete intake of the medication were significantly associated with inadequate bowel preparation analyzed by univariate analysis. It is notable that these five parameters were also the independent factors affecting inadequate bowel preparation. High-FODMAP diet, DM, and constipation were independent risk factors, whereas male sex and complete intake of the medication were independent protective factors. Cheng et al. [31] found no evidence of any association between constipation and high risk of poor bowel preparation. The same result was found in a study by Yadlapati et al. [32]. However, consistent with our previous findings [19], constipation was significantly associated with inadequate bowel preparation. DM along with gastrointestinal motility anomalies in accordance with our expectations may be independent risk factors of inadequate bowel preparation. The high-FODMAP diet plays the same role as a high-fiber diet [18] and is significantly associated with poor bowel cleansing efficacy.

The FODMAP diet was used as a dietary strategy for bowel preparation for the first time in this multicenter, prospective cohort study. The definition of FODMAP is clear, and software is available to query the FODMAP types of various foods. It is easier to understand and offers more flexible choices than the residue/fiber diet and is better tolerated than the clear fluid diet. Overall, the FODMAP dietary strategy is useful for bowel preparation.

Limitations of our study are worth noting. As this was a multicenter study, interobserver variability among endoscopists may have affected our results; however, we used the bubble score and BBPS to evaluate the quality of bowel cleansing, as have been commonly used in our previous study [17, 18] that showed excellent interobserver agreement. In this study, two experienced endoscopists scored bowel preparation in each center. To reduce subjective bias, they were trained before the study and learned the criteria of the bubble score and BBPS together. In addition, a more detailed diet classification including moderate or low FODMAP was not analyzed in the present trial; further studies need to be performed.

In conclusion, the FODMAP diet had a significant influence on the quality of the bowel preparation. The FODMAP types of various foods could be queried by software; therefore, it was flexible and referable. The high-FODMAP diet was significantly associated with poor bowel preparation. The nonhigh-FODMAP diet on the day before colonoscopy could significantly improve the quality of bowel preparation. As a reference standard for a diet regimen, whether or not the high-FODMAP diet was effective, flexible, referable, and well tolerated, it could help to provide patients with dietary guidance in bowel preparation for a Chinese population.

## Figures and Tables

**Table 1:** Baseline characteristics of the participants according to dietary group.

	High-FODMAP diet (*n* = 241)	Nonhigh-FODMAP diet (*n* = 124)	*P* value
Age, mean ± SD (years)	53.17 ± 12.76	52.64 ± 12.96	0.706
Sex, *n* (%)			0.481
Male	115 (47.7%)	64 (52.4%)	
Female	126 (52.3%)	60 (47.6%)	
Body mass index, mean ± SD (kg/m^2^)	23.70 ± 3.10	23.91 ± 3.79	0.594
Indication, *n* (%)			
Diarrhea	20 (8.3%)	16 (12.9%)	0.162
Bloating	18 (7.5%)	5 (4.0%)	0.201
Abdominal pain	36 (14.9%)	8 (6.4%)	0.018
Bleeding	16 (6.6%)	8 (6.5%)	0.945
Anemia	2 (0.8%)	0 (0.0%)	0.309
Weight loss	4 (1.7%)	2 (1.6%)	0.973
Increased CEA level	1 (0.4%)	4 (3.2%)	0.029
Change in bowel habits	4 5 (18.7%)	23 (18.5%)	0.977
Postpolypectomy	36 (14.9%)	17 (13.7%)	0.752
Health examination	49 (20.3%)	35 (28.2%)	.090
Others	14 (5.8%)	6 (4.8%)	0.700
Past history of CRC	19 (7.9%)	5 (4.0%)	0.160
Past history of polyps	18 (7.5%)	4 (3.2%)	0.107
History of abdominal surgery	65 (27.0%)	34 (27.4%)	0.927
History of gallbladder resection	11 (4.6%)	8 (6.5%)	0.442
Diabetes mellitus	15 (6.2%)	8 (6.6%)	0.932
Hypertension	54 (22.4%)	21 (16.9%)	0.220
Constipation	59 (24.5%)	24 (19.4%)	0.268
Complete intake of the medication	234 (97.1%)	121 (97.6%)	0.788

Data are presented as mean ± standard deviation or number (percentage) as appropriate; SD: standard deviation; CEA: carcinoembryonic antigen; CRC: colorectal cancer.

**Table 2:** Comparison of the two groups on colonoscopy procedure-associated parameters.

	High-FODMAP diet (*n* = 241)	Nonhigh-FODMAP diet (*n* = 124)	*P* value
Cecal intubation time, mean ± SD (minutes)	7.07 ± 5.18	5.46 ± 3.05	0.002
Withdrawal time, mean ± SD (minutes)	7.61 ± 3.97	7.48 ± 6.09	0.829
Diagnosis, *n* (%)			
Overall polyp detection rate	91 (37.8%)	37 (29.8%)	0.133
Overall adenoma detection rate	56 (23.2%)	32 (25.8%)	0.587
Overall advanced adenomas detected rate	21 (8.7%)	11 (8.9%)	0.960
Normal	118 (49.0%)	73 (58.9%)	0.073
Carcinoma	2 (0.8%)	0 (0.0%)	0.309
IBD	2 (0.8%)	1 (0.8%)	0.981
Others	28 (11.6%)	13 (10.5%)	0.745

SD: standard deviation; IBD: inflammatory bowel disease.

**Table 3:** Comparison of BBPS and bubble scores in the two groups.

	High-FODMAP diet (*n* = 241)	Nonhigh-FODMAP diet (*n* = 124)	*P* value
Bubble score	2.42 ± 1.69	1.32 ± 1.63	<0.001
Right-side colon	1.03 ± 0.71	0.65 ± 0.75	<0.001
Transverse colon	0.78 ± 0.66	0.42 ± 0.65	<0.001
Left-side colon	0.61 ± 0.62	0.26 ± 0.52	<0.001
BBPS score	6.21 ± 1.62	6.86 ± 1.37	0.001
Right-side colon	1.82 ± 0.64	2.06 ± 0.55	0.01
Transverse colon	2.09 ± 0.61	2.32 ± 0.53	<0.001
Left-side colon	2.29 ± 0.68	2.48 ± 0.61	0.007
Patients with BBPS ≥ 6 (%)	185 (76.8%)	112 (90.3%)	0.002

BBPS: Boston bowel preparation scale.

**Table 4:** Univariate analyses of risk factors for inadequate bowel preparation.

	Adequate preparation (*n* = 297)	Inadequate preparation (*n* = 68)	*P* value
Center, *n* (%)			0.139
Center 1	154 (51.9%)	42 (61.8%)	
Center 2	143 (48.1%)	26 (38.2%)	
Sex, *n* (%)			0.020
Male	137 (46.1%)	42 (61.8%)	
Female	160 (53.9%)	26 (38.2%)	
Age, mean ± SD (years)	52.51 ± 13.02	55.10 ± 11.72	0.110
BMI, mean ± SD (kg/m^2^)	23.82 ± 3.40	23.58 ± 3.11	0.576
Dietary group, *n* (%)			0.002
High-FODMAP diet	185 (62.3%)	56 (82.4%)	
Nonhigh-FODMAP diet	112 (37.7%)	12 (17.6%)	
Past history of CRC, *n* (%)	23 (7.7%)	1 (1.5%)	0.060
Past history of polyps, *n* (%)	20 (6.7%)	2 (2.9%)	0.236
History of abdominal surgery, *n* (%)	80 (26.9%)	19 (27.9%)	0.986
History of gallbladder resection, *n* (%)	16 (5.4%)	3 (4.4%)	0.744
Diabetes mellitus, *n* (%)	14 (4.7%)	9 (13.2%)	0.009
Hypertension, *n* (%)	58 (19.5%)	17 (25.0%)	0.314
Constipation, *n* (%)	59 (19.9%)	24 (35.3%)	0.006
Complete intake of the medication, *n* (%)	292 (98.3%)	63 (92.6%)	0.010

BMI: body mass index; BBPS: Boston bowel preparation scale.

**Table 5:** Multivariate logistic regression analysis of independent risk factors for inadequate bowel preparation.

Parameters	*B*	Odds ratio	95% confidence interval	*P* value
High-FODMAP diet	1.070	2.917	1.466-5.803	0.002
Male	-0.959	0.383	0.212-0.694	0.002
Diabetes mellitus	1.268	3.552	1.352-9.332	0.010
Constipation	0.927	2.528	1.361-4.698	0.003
Complete intake of the medication	-1.879	0.153	0.037-0.625	0.009

## Data Availability

The data used to support the findings of this study are included within the article.

## Abbreviations

FODMAP: Fermentable, oligosaccharides, disaccharides, monosaccharides and polyols
BBPS: Boston bowel preparation scale
ADR: Adenoma detection rate
CRC: Colorectal cancer
BMI: Body mass index.
